# Blocking transmembrane219 protein signaling inhibits autophagy and restores normal cell death

**DOI:** 10.1371/journal.pone.0218091

**Published:** 2019-06-20

**Authors:** Sean Joyce, Adel M. Nour

**Affiliations:** Department of Molecular Microbiology and Immunology, Brown University, Providence, Rhode Island, United States of America; Univerzitet u Beogradu, SERBIA

## Abstract

Autophagy plays a vital role in tumor therapy and survival of dormant tumor cells. Here we describe a novel function of a protein known as Transmembrane 219 (TM219) as an autophagy activator. TM219 is a small membrane protein expressed in all known human tissues except the thymus. We used biochemical approaches to identify calmodulin and calmodulin dependent protein kinase II as a part of TM219 protein complex. Then, we employed *in vitro* reconstitution system and fluorescence anisotropy to study the requirements of TM219 to bind calmodulin *in vitro*. We also used this system to study the effects of a synthetic peptide derived from the sequence of the short cytoplasmic tail of TM219 (SCTT) on calmodulin-TM219 receptor interactions. We conjugated SCTT peptide with a pH Low Insertion peptide (pHLIP) for optimal cellular delivery. We finally tested the effects of SCTT-pHLIP on triple negative human breast cancer cells in three dimension culture. Our data defined a novel function of TM219 protein and an efficient approach to inhibit it.

## Introduction

It is estimated that one in every eight women will develop breast cancer in USA. Among the breast cancer patients, there are 15–20% diagnosed with an invasive triple negative tumor [1] have the worst therapy outcome and have the shortest survival.

Studies have linked cancer therapy resistance to tumor dormancy, in which cancer micro-environment and macroautophagy (hereafter called ‘autophagy’) play the key roles to allow tumor cells to survive in the harsh conditions during therapy [2].

Autophagy is a general mechanism by which cytoplasmic components, including macromolecules and organelles, are directed to the endocytic pathway for degradation or secretion[3]. Autophagy has complex and highly context-dependent roles in human disease [4], including tumorigenesis where it plays the key role in tumor maintenance and progression[5–7]. This process is initiated after specific phosphorylation of an endoplasmic protein Beclin1, which is mediated by a variety of kinases [8–11]. Beclin1 phosphorylation releases the anti-apoptotic protein Bcl2 and activates the lipid kinase vacuolar protein sorting 34 (VPS34). Release of Bcl2 inhibits apoptosis during autophagy while activation of VPS34 kinase induces the autophagosome formation [12–13].

Emerging evidence has also demonstrated that autophagy plays a critical role in the survival of dormant cancer cells [14–15] that drive tumor invasion and therapy resistance [16–18]. During cancer dormancy period, autophagy is believed to provide the energy via recycling the internal cellular compartments in the lysosomes [19–20]. Moreover, autophagy activation in tumors has been linked to the evasion of the immune surveillance [21], activation of multidrug resistance pathways [22], and enhancement of DNA repair mechanisms [23]. These events are associated with the emerging of cancer stem cells or tumor dormancy and contribute to the survival of tumor cells and to their known resistance to chemotherapy and other therapeutic interventions [24–25].

Insulin-like growth factor binding protein 3 (IGFBP3) is one of 6 related proteins that bind insulin growth factor 1 and II (IGF1/II) with high affinity [26]. Early studies focused on its regulation of somatic cell growth via its ability to transport IGFs in a tertiary complex with acid labile subunit (ALS) [27]. Over the last decade or so it has become clear that IGFBP3 has a variety of other ligands and receptors [28–32]. These studies have placed IGFBP3 at the cross roads of cell death and survival [33] and have highlighted its ability to regulate apoptosis [34], DNA double strand break repair [35] and the induction of autophagy via the endoplasmic reticulum chaperone GRP78 (also called Bip) [36]. These studies have also highlighted ligand-receptor interactions between IGFBP3 and the transmembrane protein TM219 which induce caspase 8-dependent apoptosis [32], [37] though it has 17 amino acids short cytoplasmic tail lacking any homology to known cell death domains. In disagreement with the previous studies, IGFBP3, the putative ligand of TM219 protein, is upregulated after allergen challenge in asthma animal model and in patients with asthma [38]. Moreover, breast cancer patients with poor diagnosis overexpress IGFBP3 at mRNA and protein levels [39]. In keeping with the importance of IGFBP3 and TM219 in the regulation of cell survival, IGFBP3 has been shown to be dysregulated in a variety of cancers [39–43]. However, its roles in the biology of these tumors and the importance of TM219 in these responses have not been adequately defined. In addition, because TM219 only has a short 17 amino acid cytoplasmic tail that lacks a death domain, the mechanism via which IGFBP3-TM219 binding induces intracellular signaling and regulates cell death responses has not been addressed. In addition, the possibility that interventions that block IGFBP3-TM219 signaling pathways might have therapeutic utility in cancer has not been addressed.

We hypothesized that the IGFBP3-TM219 pathway plays important roles in the regulation of autophagy and the cell death in triple negative cells. We also hypothesized that these effects are mediated by the ability of TM219 to bind to a unique intracellular binding partners and that intervention in this pathway will alter autophagy and cell death response in cancer. To test these hypotheses we characterized the importance of TM219 in IGFBP3 mediated autophagy and the mechanism by which TM219 induces its effector responses. We also devised a mechanism by which the IGFBP3-TM219 pathway could be interrupted and characterized the consequences of this intervention. These studies demonstrate that TM219 is a novel activator of autophagy induced by IGFBP3. They also demonstrate that calmodulin and calcium/calmodulin dependent kinase II are downstream effector molecules in IGFBP3-TM219 responses. Lastly, they demonstrate that a therapeutic peptide based on the amino acid sequence of the short cytoplasmic tail of TM219 can block autophagy in tumor cells *in vitro* and the growth of cells in 3D culture.

## Materials and methods

### Antibodies, peptides, constructs, and cell lines

Rabbit anti-TM219 antibody was purchased from Novagen, mouse anti-TM219 was purchased from R&D systems, mouse anti-β-actin-HRP antibody from Santa Cruz biotechnology, rabbit polyclonal anti-phospho-Beclin1 from Affinity biosciences. We purchased the following rabbits antibodies from Cell signaling: Anti-calnexin, anti-LC3, anti-calmodulin, anti-caMKII and anti-CD63 antibodies.

Anti-TM219 antibody (mouse) was crosslinked to horseradish peroxidase (Thermofisher Scientific) according to the provider recommendation.

For TM219-eGFP fusion, we cloned TM219 into the N-terminal or the C-terminal of the enhanced green florescence protein (eGFP) of pEGFP-N2 vector (Addgene). We tested the expression of both constructs in Vero cells. Only construct fused with C-terminal of eGFP emits a detectable green fluorescence signal. Lamp1-monomeric red fluorescence (mRFP) and LC3-mRFP were obtained from Addgene. TM219 CRISPR/Cas9 constructs were synthesized by Genscript. We used 1 μg/ml puromycin (final concentration) for stable transfection of TM219 CRISPR/cas9 or CRISPR/cas9 construct. This concentration was chosen based on treatment of 5x10^5^ cells/ml of either Vero or MCF7 cells grown in 6 well plates with a different concentration range of purmycin (0.25–10 μg/ml). Lipofectamine2000 was used to transfect mammalian cells according to the provider recommendation (Thermofisher Scientific). We purchased specific caMKII and scrambled siRNAs from Santa Cruz biotech. For siRNAs transfection, Lipofectamin3000 was used according to the provider recommendations (Thermofisher Scientific). TM219 short cytoplasmic tail- pH low insertion peptide (SCTT-pHLIP, AEQNPIYWARYADWLFTTPLLLLDLALLVDADEGTC-s-s-CFHPRRESHWSRTRL), Cy3- pH low insertion peptide (Cy3-pHLIP) (AEQNPIYWARYADWLFTTPLLLLDLALLVDADEGTC-s-s-C(cy3), pH low insertion peptide, short cytoplasmic tail peptide of TM219 (CFHPRRESHWSRTRL) and the biotinylated short cytoplasmic tail of TM219 were synthesized by Peptide 2. MDA-MB231, MCF7, Vero, and Thp1 cells were obtained from the American type culture collections (ATCC).

### Cells surface biotinylation assay

Cells were grown in Dulbecco’s modified Eagle medium (DMEM) supplemented with 10% fetal bovine serum (FBS), 10 mM non-essential amino acids and bacterial antibiotics pen-strep (final conc. 1%). Before biotinylation, cells were washed 3 times with phosphate buffered saline (PBS) and kept on ice for 5 minutes before adding 50 μM sulfo-NHS-S~S-biotin crosslinker (Thermofisher Scientific). After adding the crossliker, the cells were kept on ice for an additional 30 minutes followed by 3 times washing with cold PBS. Cells were lysed with radio-immunoprecipitation assay buffer (RIPA) (10 mM Tris-Cl (pH 8.0) 1 mM EDTA. 1% Triton X-100 (TX100). 0.1% sodium deoxycholate, 0.1% SDS. 140 mM NaCl) supplemented with protease inhibitor cocktail and subjected to high speed centrifugation 10,000 RPM for 10 minutes at 4°C). Clear lysates were incubated with streptavidin agarose beads at 4°C for 30 minutes. The protein bound beads were collected by brief centrifugation at 1500 RPM for 30 second at 4°C. The beads were washed 3 times with the same lysis buffer and eluted with protein loading buffer supplemented with 10 mM dithiothreitol (dTT). A fraction of the lysates, flow through, and the eluate were quantified with BCA kit (Thermofisher scientific). 20 μg/μl of each fraction was subjected to immunoblot using anti-TM219 antibody. As a negative control, we used TM219 knockout Vero cells (Data are not shown).

### Immunopurification & immunoblotting assays

For immunoprecipitation of TM219 complex, we treated 6X10^6^ THP1 cells grown in Roswell park memorial institute 1640 medium (PRIM) without serum with 1 μM IGFBP3 for 1 hour. Cells were lysed with immunoprecipitation buffer (1% NP-40, 150 mM NaCl, and 50 mM Tris pH 8.4 in the presence of 2 X protease inhibitors (Roche)). Crosslinking of mouse anti-TM219 antibodies to protein A-Sepharose was performed as previously described [44]. Briefly, 1 mg of the antibody was incubated with 1 ml protein A-Sepharose beads (Thermofisher scientific), with continuous mixing at 4°C for 2 h. The beads were then centrifuged and washed five times with PBS and once with 200 mM sodium borate, pH 9. Equal volumes of 40 mM dimethylsuberimidate (Thermofisher scientific) in 200 mM sodium borate were mixed with the beads, and the suspension was incubated, with continuous mixing, at room temperature for 30 min. The reaction was terminated by addition of equal volumes of 400 mM ethanolamine, and the sample was incubated for an additional 30 min. The beads were packed into a 1-cm-diameter column (Bio-RAD) and washed three times with 2 bed volumes of PBS and once with 4 bed volumes of 100 mM glycine-HCl at pH 2.4 to remove non-cross-linked antibodies. Finally, the cross-linked antibody beads were washed with PBS until the pH reached 7 and stored at 4°C in PBS containing 0.02% sodium azide. The crosslinked antibodies were packed into columns (Bio-RAD) and used to immunoaffinity purification of the TM219 protein-associated complex. As a control, we crosslinked total immunoglobulin G (IgG) (Sigma-Alderish) from pre-immunized mice (control Beads,) to ensure the specificity of anti-TM219 antibody in pulling down the TM219 receptor complex. Samples were eluted from the beads using the sample elution a sample loading buffer without reducing agent. Reducing agent 1mM DTT was added to the eluate before the heat denaturation step (95°C for 5 minutes). Samples were resolved on 4–20% sodium dodecyl sulfate-denature polyacrylamide gel electrophoresis (SDS-dPAGE) (Bio-RAD), and electroblotted to polyvinylidene difluoride membranes (Bio-RAD) according to the manufacturer’s protocol.

For phosphorylation assay of Beclin1 protein, we treated 6x10^6^ MCF7 cells grown in DMEM serum free medium with 1 μM IGFBP3. Lysates were prepared in RIPA buffer in presence of 2X protease and phosphatase inhibitors (Thermofisher Scientific). Lysates were clarified by centrifugation at 10,000 rpm for 30 minutes at 4°C. Finally, the blotted membranes were probed with anti-TM219, LC3, calmodulin, phospho-beclin1, or β-actin. To quantify the ratio between LC3-II and LC3-I, we used an image densitometry method using ImageJ software [45].

### Microscopy

Immunostaining was performed as described previously (44). Briefly, cells were grown on a coverslip overnight at 5×10^5^ cells/ml. 1 μl of Hoechst stain (10 μg/μl) was added to the cells for 10 min at 37°C. For tracking TM219 short cytoplasmic peptide assay, we pretreated the cells with the peptides at concentration of 0, 25, or 250 nM, then added 1 μM IGFBP3 for an hour in DMEM serum free medium. Cells were fixed with 4% paraformaldehyde and permeabilized with 1% triton x100. Cells were then blocked overnight with 10% fetal bovine serum (FBS) in PBS. Finally, cells were stained with streptavidin conjugated to Alex555 (Thermofisher Scientific) for 30 minutes in the same blocking buffer. For immunostaining, cells were stained with the primary antibody according to the manufacturer’s recommendation in the same blocking buffer. The cells were washed 10 times with PBS. Cells were blocked for 1 h with 10% FBS and then stained with secondary antibody conjugated to fluorescence dye (Thermofisher Scientific). As controls, we used secondary antibodies alone. Cells were washed 10 times with PBS and mounted with fluoromount G (Microscopic Science) before examination.

We stained the intact cellular mitochondria using TMRM dye according to the provider recommendations (Thermofisher Scientific). To evaluate the cell membrane permeability in 3D culture, we used membrane impermeable dye propodeum iodide (PI), and the membrane permeable dye (Hoechst dye) to stain the cell nuclei according to the manufacturer recommendations (Thermofisher Scientific). For autophagy efflux assay, we used chloroquine (Sigma-Alderish) (CQ) at final concentration of 200 μM as described previously [46]. Collected images were analyzed with ImageJ software.

### Protein purification

Calmodulin was cloned from cDNA library prepared from human Thp1 cells. Briefly, total RNA was extracted from Thp1 cells using triazol method (Thermofisher Scientific) and reverse transcribed using oligo.(dT)_12-18_ primer and superscript IV reverse transcriptase (Thermofisher Scientific) according the provider recommendations. One tenth of the reverse transcribed RNA (2 μl) was subjected to PCR amplification using specific primers derived from human calmodulin mRNA CalF (ATATAATAGGATCCATGGCTGACCAGCTGACTGAGGAGCAG) and CalR (ATATAATAGCGGCCGCCTTTGCAGTCATCATCTGTACAAACTC). The PCR product, 560 base pairs, was subjected to DNA sequencing before cloning into bacterial pET28 expression vector. A positive clone was used to express calmodulin at small scale (4 ml) by adding 0.1mM isopropyl β-thiogalactoside (IPTG) induction for 6 hours at 4°C to Luria Broth (LB) medium inoculated with transformed *E*.*coli* (Rosetta strain) at OD600 = 0.6. Protein expression was confirmed using Western immunoblot hybridization using anti-calmodulin antibody before scaling up.

To scale up, culture of transformed Rosetta strain of *E*.*coli* with human calmodulin construct was induced with 0.1 mM IPTG overnight (about 16 hours) at 4°C when the cells reached OD_600_ = 0.6. Cell lysis was prepared using 150 mM NaCl, 20 mM Tris pH 7.4 and French cell pressure in presence of protease inhibitor cocktail. 25 μg/ml RNase and 10U/ml DNase (Sigma-Alderish) were added to the lysate. Before applying to the phenyl sepharose column (GE healthcare), calcium chloride was added to final concentration of 5 mM. Column was equilibrated with the same lysis buffer containing 5 mM calcium chloride and washed with the same buffer. The protein was eluted with 20 mM Tris pH 7.4, 150 mM NaCl and 1 mM EGTA. The eluate was dialyzed against PBS buffer using 10 KD cut off dialysis bag (Thermofisher scientific). For removing the contaminants that co-eluted with calmodulin, we used anion exchanger column (GE healthcare). The protein was eluted from the column resin with gradient concentration of 0–100% NaCl in 10mM Tris pH 7.4. Purified calmodulin containing fractions were combined and concentrated using ultracentrifugation tube with cut of 10 KD (Millipore-Sigma). Purity and integrity of calmodulin were checked by size exclusion chromatography (S200 column) and 4–20% SDS-dPAGE & *Coomassie* stain. Transmembrane219 cloning, expression, purification, detergent screening, and size exclusion chromatography were described previously [47].

### TM219 nanodisc & fluorescence anisotropy assays

Reconstituted TM219 into 13 nm nanodisc and labeling of our purified calmodulin or IGFBP3 (R&D systems) with BODIPY-TRM-X-succinimidyl ester dye were described previously [47]. For peptide inhibition assay, we mixed 1μM IGFBP3, 40 μM BODIPY-TRM-X-succinimidyl ester dye labelled calmodulin, 1 mM calcium chloride, and 0.5 μM TM219 nanodisc. Serial dilution of the TM219 short cytoplasmic peptide (1pM-10mM) was used. Fluorescence anisotropy assays were done in triplicates and data represent an average of three independent experiments. Each sample was run in triplicates and an average of 60 readings was used for calculation. Binding constants (KDs) or inhibition constant (Ki) were determined with Origin 7 (OriginLab) by fitting the data as described previously [48].

### Measuring calcium in cell lysate

We used a colorimetric kit to measure the increase in the cytosolic calcium in MCF7 cells according to the provider recommendations (Abcam). MCF7 cells were grown in completed DMEM medium at density of 5x10^5^ cells/ml in 6 well plates. We compared the effects of 1μM IGFBP3 on the wild type, TM219 knockout and untreated MCF7 cells after 5 minutes in serum free medium. As a positive control we used 10 mM ionomycin (Thermofishter Scientific). As a negative control we pretreated MCF7 cells with 25 mM BAPTA for 30 minutes (Thermofisher Scientific). Experiments were run in triplicate.

### Pull-down assay & mass spectrometry analysis

To identify proteins interacting with TM219 short cytoplasmic tail, we synthesized an N-terminal biotinylated polypeptide based on the amino acids sequence of the TM219 cytoplasmic tail (Peptide2). We inserted a hydrophilic linker between the biotin and the peptide. Soluble lysate of Thp1 cells was prepared in 140 mM NaCl, 1% TritonX100 and 50 mM Tris pH 7.4 in presence of 2X protease and phosphatase inhibitors cocktails at 4°C. After that the peptide was incubated with the soluble lysate for 4 hours at 4°C. Then, streptavidin agarose beads were added to the mix to purify the proteins bound to the biotinylated polypeptide. As a control, we mixed THP1 soluble lysate with streptavidin beads alone. The agarose beads were collected and washed six times with the same binding buffer before eluting the beads with 0.1 mM glycine HCl pH 2.3. We resolved the eluate on 4–20% SDS-dPAGE and stained the gel with *Coomassie* stain. We cut the major protein visible bands from the gel along with a piece of gel far away from the running sample as a negative control. We also sent the negative control and the eluted samples in solution for identification. LC Ms/Ms mass spectrometric analysis has been done at Yale Keck proteomic facility.

### Three dimension cell culture

We used soft-agar method [49] to study the effects of TM219 ligand IGFBP3 on the growth and survival MDA-MB231 cells in presence or absence of the short cytoplasmic tail of TM219 peptide. Briefly, 5x10^5^ cells were grown in 0.5% low melting agarose for 5 days. Cells were treated with 1μM IGFBP3 (R&D systems) and 250 nM SCTT peptide for an additional 9 days. Spheroids were imaged using the phase contrast microscopy. The changes in the spheroids radius and volume were calculated based on the assumption that the spheroids are spherical. Data collected from 100 spheroids. To determine the ratio between the dead and living cells, we recovered the spheroid cells from the agarose by treatment with 6 M sodium iodide at 37°C for 2–5 minutes and spin down briefly (1500 rpm, for 5 minutes at 4°C). Recovered spheroids were treated with 0.025% Trypsin for 5 min. at 37°C and subjected to trypan blue staining to assess the living and dead cells. Samples were run in triplicates, and the average was taken for every sample.

## Results

### Transmembrane 219 has two putative cytoplasmic ER retention signals

**Fig 1:**
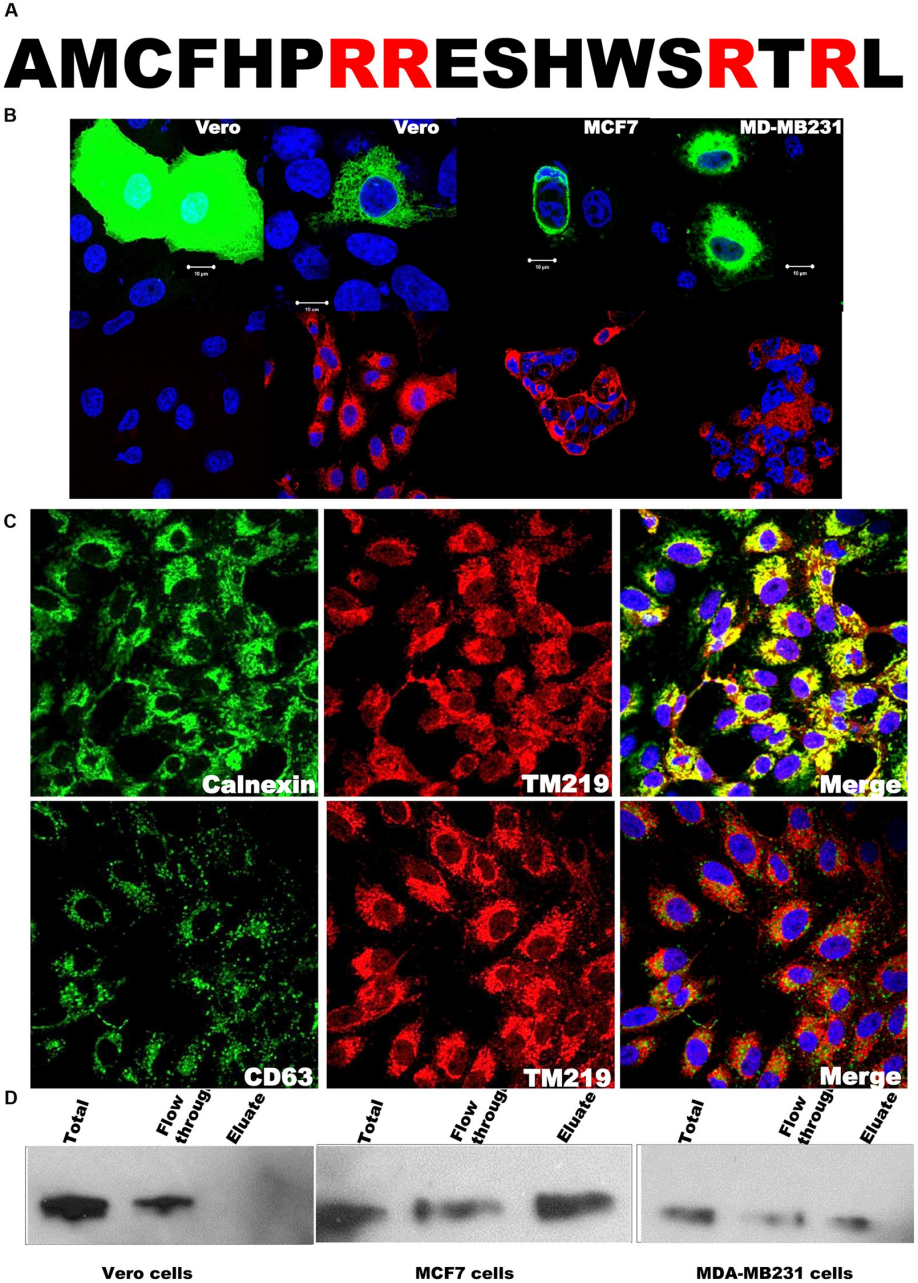
Transmembrane219 (TM219) resides in the perinuclear and on the cell surface of some human breast cancer cells. **A**- The amino acid sequence of the cytoplasmic tail of TM219 protein has two predicted endoplasmic reticulum (ER) retention di-arginine motifs. Presence of RR and RTR di-arginine in the truncated cytoplasmic domain suggested that TM219 protein requires posttranslational modification and assembly in order to exit the ER compartment. **B**- Vero, MCF7 or MDA-MB231 cells were transfected with TM219-eGFP construct. As a control, Vero cells were transfected with eGFP alone. After 48 hours of transfection, cells were fixed with paraformaldehyde and examined with fluorescence microscopy. Cells overexpressing TM219-eGFP manifested the characteristic pattern of perinuclear distributed proteins. The same cells were immunostained for the endogenous TM219 protein. TM219 knockout Vero cells were used as a control. Nuclei were stained with the Hoechst dye (blue). The pattern of GFP-TM219 distribution was consistent with the endogenous TM219 immunostaining. **C**- Vero, MCF7 or MDA-MB231 cells were immunostained with anti-TM219 antibody followed by immunostaining with either anti-calnexin or anti-CD63 antibodies. Endogenous TM219 was overlapped with the ER molecular chaperon calnexin and partially overlapped with the late endosomal CD63 protein. **D**- Cell surface biotinylation assay was used to study the surface expression of TM219 protein in Vero, MCF7 or MDA-MB231 cells. A lysine crosslinker conjugated to biotin was used to crosslink the cell surface proteins. After that, the soluble cell lysates were passed through streptavidin conjugated agarose beads to capture the biotinylated proteins. Finally, 20 μg/μl of the total protein of the lysate, flow through, and the eluate were resolved on 4–20% SDS dPAGE and immunoprobed with anti-TM219 antibody. A detectable fraction of TM219 protein was only on the surface of MCF7 and MDA-MB231 cells. Experiments were run in triplicate for each cell line.

Transmembrane 219 (also known as Insulin-like growth factor binding protein 3(IGFBP3) receptor. Tmem219 or TM219) is a small single span membrane protein with a calculated molecular weight ~ 25 KDa. This protein has a 17 amino acid cytoplasmic tail with two predicted di-arginine motifs (Fig 1A), a motif that retains membrane proteins in the endoplasmic reticulum (ER) compartment before assembly and trafficking to the right membranous compartments [50–51]. To study the subcellular localization of TM219 protein, we fused TM219 with the eGFP as described in materials and methods. We expressed this construct in different epithelial cell lines such as Vero cells, MCF7 cells, or MDA-MB231 cells. Expression of TM219- eGFP in non-malignant cells such as Vero cells showed a perinuclear characteristic network pattern of either mitochondria or ER localized proteins (Fig 1B, upper left panel). However, a detectable fraction of this protein was also on plasma membrane and endoplasmic reticulum of the malignant epithelial cells MCF7 and MDA-MB231 cells (Fig 1B, middle and right panels). To confirm these data, we used anti-TM219 antibodies to immunostain the endogenous TM219 protein. The pattern of endogenous TM219 protein staining was consistent with the expression of TM219-eGFP (Fig 1B, lower panels). As a control for the endogenous staining of TM219, we used TM219 knockout Vero cells (Fig 1B, lower left panel). Moreover, we expressed eGFP alone in Vero cells (Fig 1B, upper left panel). We also tested for co-localization of TM219 with ER molecular chaperon protein Calnexin, late endosomal membrane protein CD63, lysosomal protein Lamp1, and the intact mitochondria, as judged by the uptake of TMRM dye. TM219 protein was localized mainly in the perinuclear region and its distrubtion pattern overlapped with the late endosomal/lysosomal proteins (Fig 1C & S1 Fig).

Because of the difficulty to find a good membrane protein marker, we optimized a cell surface biotinylation assay as described in materials and methods to study TM219 surface expression. Our data revealed that both MCF7 and MDA-MB231 cells, unlike Vero cells, expressed detectable TM219 protein on their cell surface (Fig 1D). These data collectively suggested that TM219 protein is expressed and retained in the endoplasmic reticulum. Future studies are required to explain the mechanism of assembly and trafficking of TM219 protein.

### TM219 signaling mediated by IGFBP3 activates autophagy

**Fig 2:**
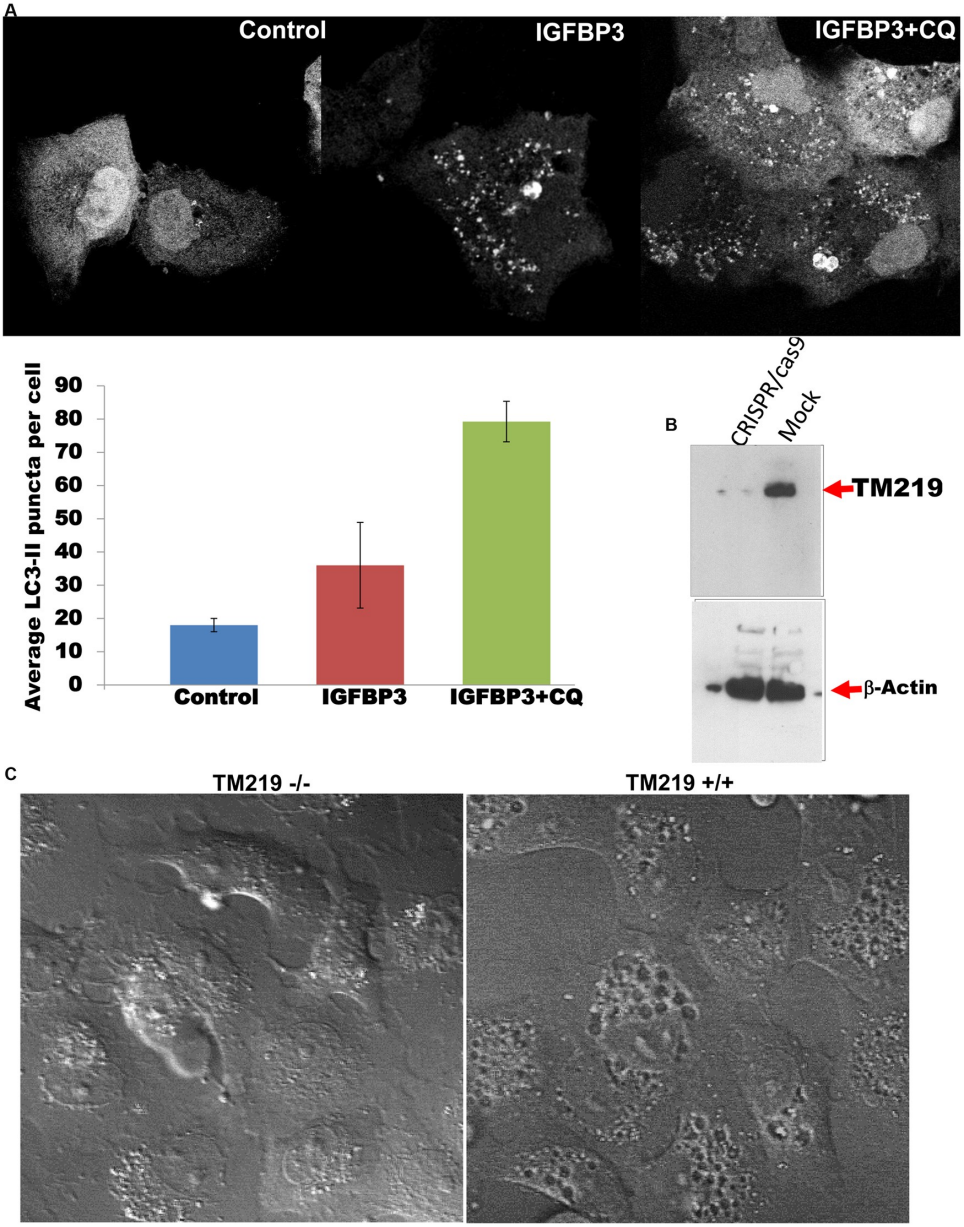
IGFBP3 mediated by TM219 receptor activates autophagy. **A**- Vero cells were transfected with LC3-mRFP and treated with of 1 μM IGFPB3 for an hour in DMEM serum free medium showed a notable increase in processing of LC3 protein (as indicated by the increase in number of LC3-II punctate staining pattern) relative to untreated control cells transfected with mRFP-LC3 construct. As an additional control, Vero cells transfected with LC3-mRFP were treated with 200 μM chloroquine (CQ), an autophagy flux inhibitor, and 1 μM IGFBP3. Data were presented as the mean of LC3-II punctate staining pattern +/- standard error of the mean (SEM). Cells treated with IGFBP3 demonstrated significant increase in number of LCII punctate stain and presence of CQ accumulates LCII positive vesicles in the cytoplasm of Vero cells. **B**- Vero cells were transfected with either TM219RNAg-CRISPR/cas9 construct or with CRISPR/cas9 construct alone were selected with 1 μg/ml puromycin. TM219 gene inactivation was confirmed by immunoblotting using specific anti-TM219 antibody. The same lysate was also immunoprobed with anti-β-actin. **C**- TM219 knockout and wild type Vero cells were treated with 1 μM IGFBP3 for an hour in serum free medium. Then, the cells were fixed with paraformaldehyde and examined DIC microscopy. TM219 wild type Vero cells demonstrated larger internal vacuoles after treatment with IGFBP3 compared to TM219 knockout Vero cells.

Previous studies described TM219 function a novel cell death receptor in human breast cancer and asthma [32], [37]. However, different independent studies reported upregulation of its ligand IGFBP3 in the same human diseases [38–39]. In order to address the function of TM219 protein that could explain the previous studies, we compared the responses of Vero cells in which TM219 was overexpressed as an eGFP fusion protein and cells in which only eGFP was expressed. Cells overexpressing TM219-eGFP appeared to be healthy with intact nuclei for two days post transfection. Interestingly, the cells also overexpressing TM219-eGFP manifest clearly perceptible large vacuoles, presumably the autophagosome, while the cells that overexpressed just eGFP did not (S2A Fig). We next immunoprobed lysates from cells overexpressing TM219-eGFP and cells overexpressing eGFP alone for hallmark of programmed cell death procaspase 3 pathways. Neither Vero cells transfected with TM219-eGFP nor the cells transfected with eGFP alone processed procaspase 3 after 48 hours of transfection (S2 Fig). In contrast these cell populations differed drastically in the processing of the characteristic protein marker of autophagy activation LC3 (S2 Fig). To study the effects of IGFBP3 on LC3 processing, we transfected Vero cells with LC3-monomeric red fluorescence (LC3-mRFP) and tested for the effects of IGFBP3 treatment on these cells. Cells transfected with LC3-mRFP and treated with IGFBP3 demonstrated a significant increase in processing of LC3, as indicated by the LC3-II punctate staining (Fig 2A). However, this punctate stain was accumulated in the cytoplasm of the cells pretreated with inhibition dose of chloroquine (200 μM), an agent that commonly use to block autophagosome fusion with the lysosome, after treatment with 1 μM IGFBP3 (Fig 2A, right panel).

In order to address the role of TM219 in autophagy activation mediated by IGFBP3, we inactivated TM219 protein from the genome of Vero and human primary breast adenocarcinoma epithelial MCF7 cells using TM219 RNA guide-CRSPR/Cas9 to confirm the importance of TM219 protein in this process. We confirmed the inactivation of TM219 by Western blot hybridization analysis (Fig 2B) and immunostaining (Fig 1B, lower left panel). After that we compared these cells to cells treated with the same CRISPR/CAS9 without an RNA guide sequence. When treated with 1μM of IGFBP3 protein and analyzed with differential interference contrast microscopy (DIC), wild type Vero cells showed larger internal vacuoles, presumably the autophagosome, when compared to TM219 knockout cells (Fig 2C). These data collectively indicated that IGFBP3 mediated by TM219 activates autophagy in a primary malignant breast cancer MCF7 and some non-malignant epithelial cells such as Vero cells.

### Identification of calmodulin as an interacting partner of TM219 short cytoplasmic domain

**Fig 3:**
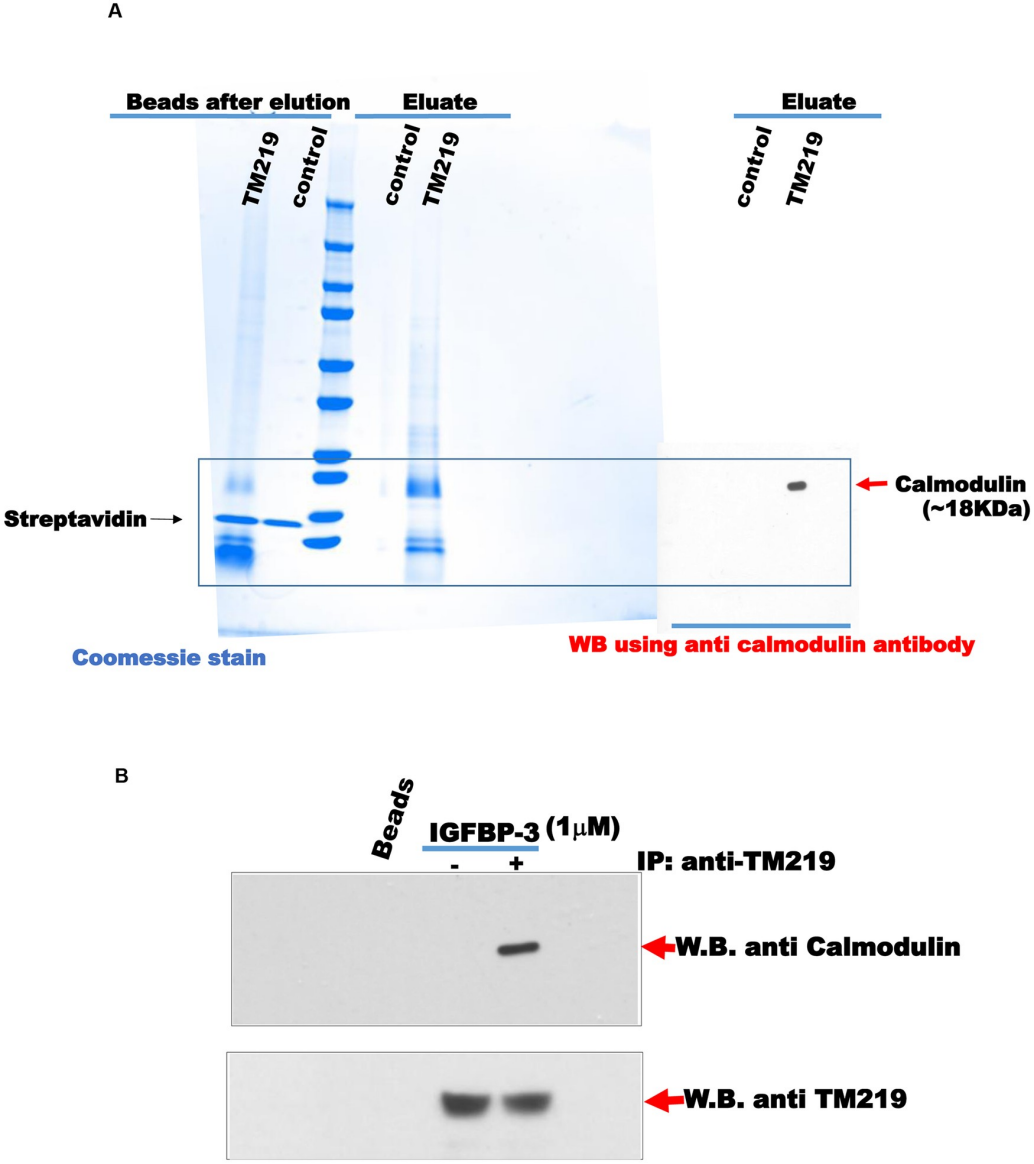
Identification of calmodulin as an interacting protein partner of the cytoplasmic domain of TM219 protein. **A**-Biotinylated form of the shot cytoplasmic domain of TM219 protein was used to purify TM219 interacting proteins form THP1 soluble cell lysate as described in materials and methods. The proteins that interact with the biotinylated peptide were captured using streptavidin conjugated beads. LC-Ms/MS mass spectrometric analysis was employed to identify the eluted proteins. *Coomassie* stain was used to monitor the eluates from both the streptavidin control beads and the biotinylated form of the short cytoplasmic domain of TM219 bound beads. Calmodulin was the highest predominant peptides identified by LC Ms/Ms. Protein eluates from both control beads and biotinylated short cytoplasmic domain of TM219 were subjected to immunoblot with anti-human calmodulin, right panel. **B**-THP1 cells were either treated or untreated with 1 μM IGFBP3 in DMEM serum free medium for an hour and subjected to an affinity purification of the TM219 receptor complex using crosslinked anti-TM219 antibodies to protein A conjugated beads. Eluates from the control normal antibody conjugated-protein A beads and anti-TM219 antibodies-protein A beads were probed with anti-calmodulin and anti-TM219 antibodies respectively.

TM219 protein has a truncated cytoplasmic domain, 17 amino acids, lacks any homology to known caspase activating domains. This short cytoplasmic tail also does not justify how TM219 activates autophagy or any other signal pathways. Thus, we hypothesized that the effects of TM219 described above are mediated by additional protein(s) that interact with TM219 receptor. In order to identify these proteins, we synthesized a biotinylated peptide based on the sequence of the TM219 cytoplasmic tail. We separated the biotin molecule from the N-terminal of the peptide via a hydrophilic linker. We then mixed this peptide with lysates from the human monocyte cells THP1 and used streptavidin-agarose beads to purify the interacting proteins with the short peptide. After washing extensively, the proteins bound to the peptide were eluted with 10 mM glycine HCl at pH 2.3 and identified by LC-MS/MS mass spectroscopy (Fig 3A and Table 1).

**Table 1:** TM219 short cytoplasmic tail interacting proteins identified by LC MS/MS mass spectrometric analysis.

Identified protein	Function (s)
Calmodulin	Signaling
Cofillin1	Actin modulating protein (Signaling)
ARF1	Small guanine nucleotide-binding protein
GRP78	ER molecular chaperon (protein folding/degradation)
C1QPB	Complement binding protein
14-3-3	Adaptor protein (Signaling)
COP-E	Coatomer subunit Epson

One of the abundant peptides identified by mass spectrometry was for human calmodulin. To confirm the interaction between calmodulin and the TM219 cytoplasmic tail peptide, we immuno-probed the purified complex with anti-human calmodulin antibody. These western blot evaluations demonstrated that calmodulin was in the pull-down proteins that interacted with TM219 peptide but was not in the pull-downs from the control streptavidin-agarose beads (Fig 3A). Further confirmation was obtained by co-immunoprecipitation assays with lysates from THP-1 cells treated with IGFBP3 in which immunoprecipitation with anti-TM219 consistently brought down calmodulin (Fig 3B). These data collectively indicate that IGFBP3 treatment triggers a complex formation between TM219 calmodulin.

### TM219-calmodulin complex requires presence of IGFBP3 protein and calcium *in vitro*

**Fig 4:**
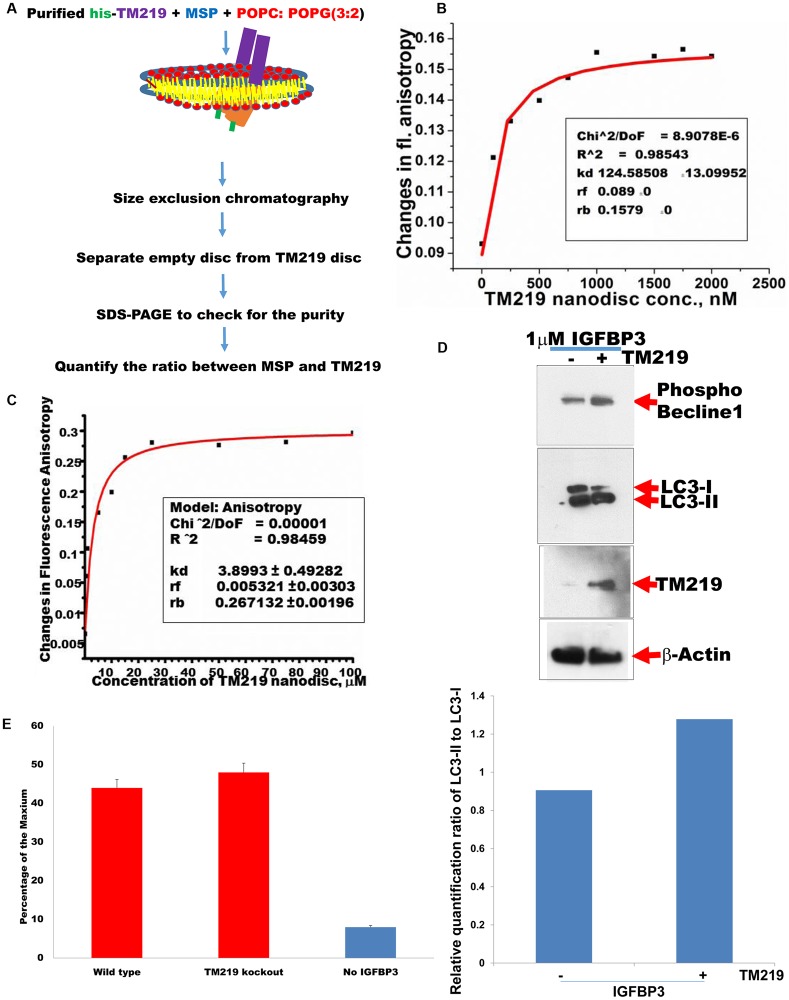
TM219 protein recruits calmodulin in presence of IGFBP3 protein and calcium. **A**-Purified TM219 protein was reconstituted into 13nm nanodisc and subjected to fluorescence anisotropy binding assay using either BODIPY-TRM-X-succinimidyl ester labelled IGFBP3 or BODIPY-TRM-X-succinimidyl ester labelled calmodulin. **B**- BODIPY-TRM-X-succinimidyl ester labelled IGFBP3 was used to measure the changes in fluorescence anisotropy using different concentrations of TM219 nanodisc (0–2500 nM). TM219 binds to labelled IGFBP3 with an affinity constant 125 nM. **C**-BODIPY-TRM-X-succinimidyl ester labeled calmodulin was tested for binding to various concentrations of TM219 nanodisc (0–100 μM) in presence and absence of IGFBP3 and calcium. TM219 nanodsic only binds to calmodulin in presence of both calcium and IGFBP3 with an affinity constant 3.9 μM. **D**- TM219 gene was inactivated from the genome of MCF7 cells using TM219RNAg-CRISPR/cas9. As a control we transfected wild type MCF7 cells with CRISPR/cas9 construct without an RNAg sequence. After 3 weeks of selection with puromycin, we treated puromycin resistant MCF7 cells with 1 μM IGFBP3 for an hour in serum free medium. We immunoprobed lysates from both TM219 wild type and TM219 knockout MCF7 cells with anti-phospho-beclin1, anti-LC3, anti-TM219 or anti-β-actin. The relative quantification ratio between LC3-II and LC3-I was measured using ImageJ software as described in materials and methods. **E**- Wild type and TM219 knockout MCF7 cells were treated with 1 μM IGFBP3 for 5 minutes. Changes in cytosolic calcium were measured as described in materials and methods. As a control, MCF7 untreated with IGFBP3 was used a control. MCF7 cells treated with 10 mM ionomycin was used as a positive control while MCF7 cells treated 25 mM BAPTA was used as a negative control. The relative calcium concentration was calculated as a percentage of the reading of the Ionomycin treated cell lysate. All readings were subtracted from the reading of the BAPTA treated cell lysate. Data were presented as the mean with error bar of SEM. IGFBP3 triggered elevation of the cytosolic calcium in MCF7 independent of TM219 receptor.

We previously reconstituted TM219 protein into 13 nm lipid bilayer [47], the nanodisc [52] and used fluorescence anisotropy assay [53] to study TM219 *in vitro*. Fig 4A summarizes the steps of TM219 reconstitution into 13 nm nanodisc.

In these studies, we used the same approach to measure the binding affinity of the labelled IGFBP3 and TM219 nanodisc. As a control, we tested for the binding of labelled IGFBP3 to the empty nanodisc to ensure that IGFBP3 protein is bound specifically to TM219 protein, not to the other nanodisc components (S4 Fig). These data indicated that IGFBP3 binds specifically to TM219 *in vitro* with an affinity constant (KD) ~125 nM (Fig 4B).

Next, we characterized the requirements for TM219 binding to calmodulin *in vitro*. We cloned human calmodulin from total RNA extracted from Thp1 cells and expressed it in *E*.*coli* as described in Materials & Methods. We purified calmodulin using hydrophobic interaction column, the anion exchanger, and the size exclusion chromatography (S3 Fig). We tested the interaction between the purified calmodulin and the TM219 nanodisc in presence of the IGFBP3. Labelled calmodulin did not bind to IGFBP3-TM219 nanodisc complex (S4 Fig). Since calmodulin contains 4 EF motifs that binds to 4 molecules of calcium in order to change its conformation [54], these experiments were repeated in the presence of 1 mM CaCl_2_, and in absence of IGFBP3. Again, we did not observe significant binding to the TM219 nanodisc (S4 Fig). In contrast, calmodulin bound to TM219 nanodisc with a KD of 4 μM in presence of calcium and IGFBP3 (Fig 4C). As an additional control, we tested the binding of calmodulin in presence of calcium and IGFBP3 with the empty nanodisc. No specific binding was detected between calmodulin the empty nanodisc (S4 Fig). These studies demonstrate that the binding of TM219 to calmodulin requires calcium ions and a ligand, in this case IGFBP3 protein. They also suggest that the TM219 protein may change its conformation upon binding to its ligand in order to bind to calmodulin bound to calcium ions. Lastly, the relatively weak binding of calmodulin to the TM219 nanodisc in the presence of calcium also suggests that higher order oligomerization of the receptor may be required for optimal binding to its downstream proteins.

### IGFBP3 treatment elevates intracellular calcium independent of TM219 receptor

Previous studies indicated that IGFBP3 independent on IGF or heparin activates intracellular rapid release of intracellular calcium in MCF7 cells [55], however the identity of the receptor that mediates this action is unknown. We first confirmed that IGFBP3 mediated by TM219 receptor activates autophagy in MCF7 cells (Fig 4D). After that we compared the effects of IGFBP3 on elevation of intracellular calcium in these cells. Cells treated with 1 μM IGFBP3 after 5 minutes in serum free medium showed relatively higher signal of calcium relative to untreated MCF7 cells (Fig 4E). However, no significant difference was observed when we compared the effects of IGFBP3 on TM219 knockout and wild type MCF7 cells, indicating that IGFBP3 protein triggers intracellular calcium elevation independent of TM219 receptor (Fig 4E).

### Calcium/Calmodulin dependent protein kinase II is associated with IGFBP3-TM219 signaling complex

**Fig 5:**
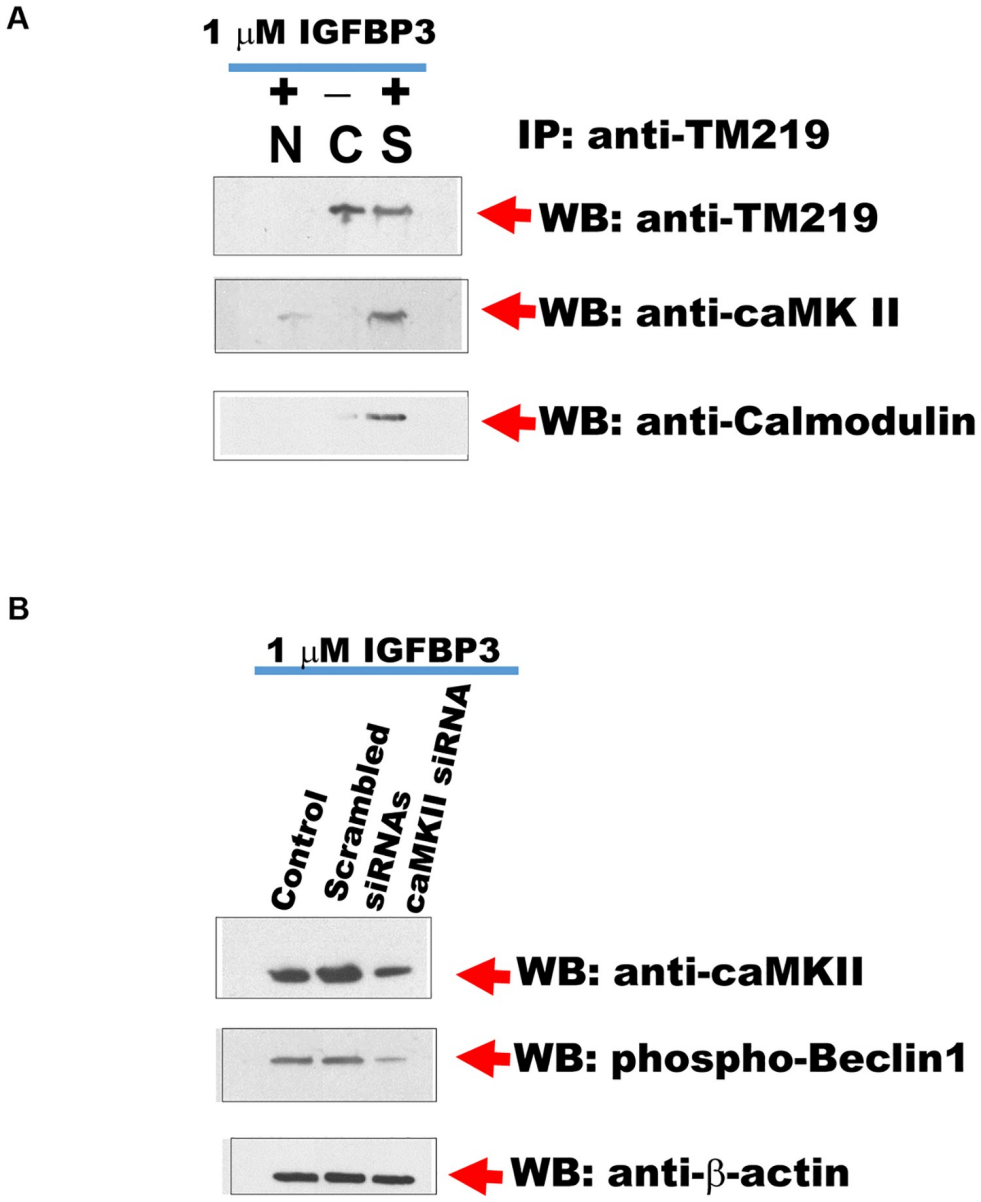
Calcium/Calmodulin dependent kinase II is associated with TM219 complex in MCF7 treated with IGFBP3. **A**-Anti-TM219 antibody crosslinked to agarose beads and packed into a column was used to purify TM219 associated proteins from MCF7 cells treated (S) or untreated (C) with 1 μM IGFBP3. As a control we used normal mouse antibodies (N) conjugated to agarose beads. Eluted proteins were immunoprobed with anti-CaMKII antibody. Purified proteins from cells treated with IGFBP3 reacted specifically with anti-CaMKII antibody. These purified proteins were associated with also associated with calmodulin. **B**-Knockdown of CaMK II transcript using specific siRNAs diminished the phospho-beclin-1 signal in cells treated with IGFBP3 compared to MCF7 cells transfected with scrambled siRNAs or wild types.

There are many known protein kinases activate autophagy via calcium/camodulin [56–60]. We immunopurified TM219 protein complexes from MCF7 cells treated or untreated with 1 μM IGFBP3 protein as described in Materials and Methods. As a control we used normal mouse antibodies conjugated beads as a negative control. We then immunoprobed the purified proteins with anti-DAP1 kinase, anti-caMKI, anti-caMKII or anti-CaMKIV antibodies. Surprisingly, anti-caMK IIα reacted specifically only with the purified proteins from MCF7 treated with IGFBP3 (Fig 5A). To confirm CaMK II is a part of TM219 complex, we also immunoprobed the purified proteins with anti-calmodulin antibody. Data revealed that TM219 protein recruits calmodulin and calmodulin dependent kinase II after treatment with IGFBP3. To confirm that TM219 activates autophagy mediated by CaMKII, we knocked down CaMKII using specific siRNA in MCF7 cells. We then tested for Beclin1 phosphorylation after treatment with IGFBP3 in cells transfected with CaMKII specific siRNAs compared to cells either transfected with scrambled siRNAs or untransfected control MCF7 cells. We used in this experiment anti-phospho-beclin1 (Ser 90/93/96) as an indication for autophagy activation because of the high background of LC3 processing that we observed in these cells (Fig 4D). Data indicated that reduction of CaMKII is correlated significantly to the reduction of Beclin1 phosphorylation in MCF7 cells (Fig 5B). When viewed in combination, these observations suggested that IGFBP3 activates autophagy via TM219 receptor and triggers an increase in cytosolic calcium via another unknown receptor. They also indicated that IGFBP3-TM219 signaling module phosphorylates Beclin1 to activate autophagy.

### TM219 short cytoplasmic protein tail synthetic peptide disrupts the interaction between calmodulin and TM219 protein

**Fig 6:**
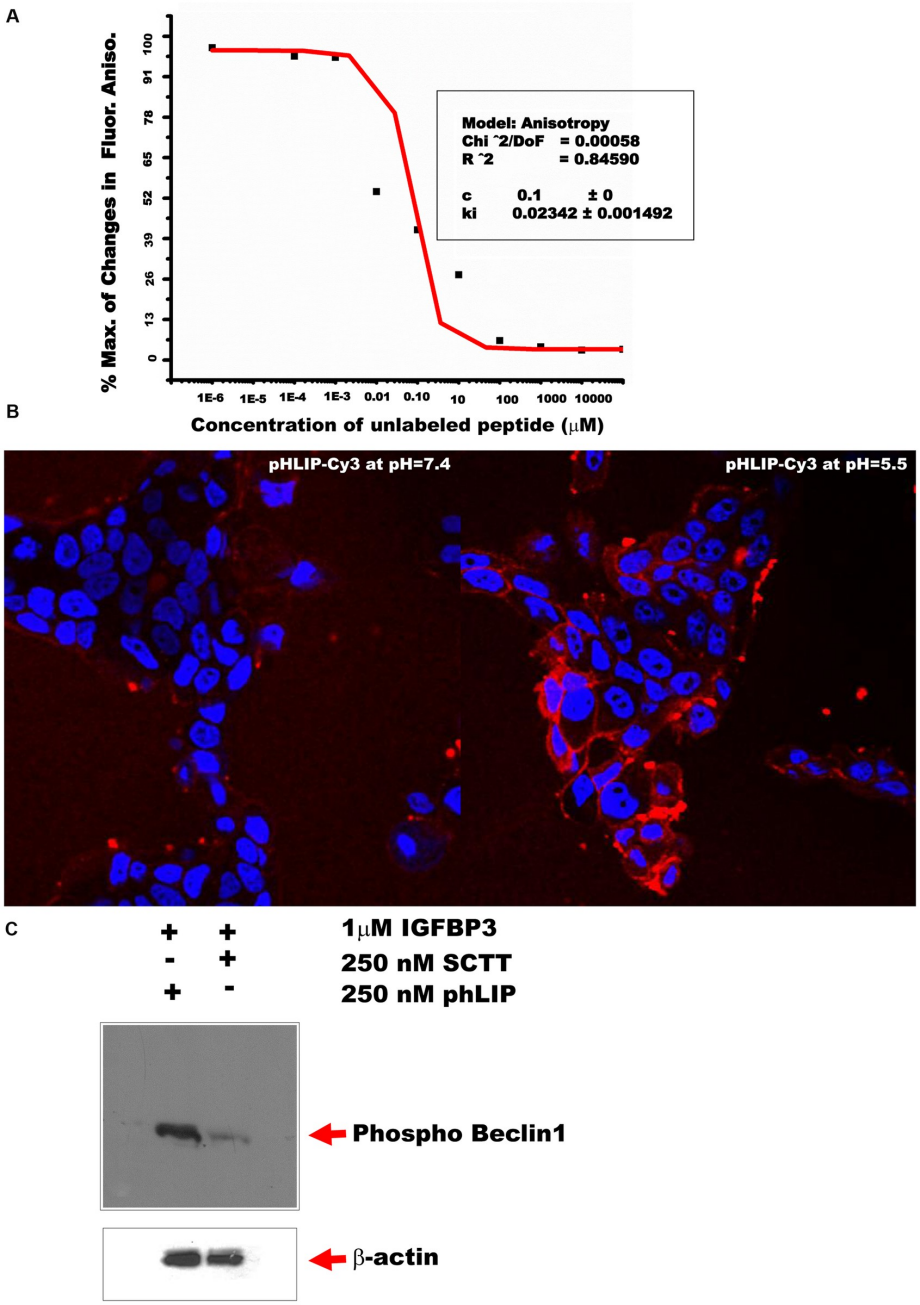
TM219 short cytoplasmic protein tail peptide disrupts the interaction between calmodulin and TM219 protein. **A**- We assembled the tetrapartate complex of TM219 nanodisc by mixing TM219 nanodisc, IGFBP3, BODIPY-TRM-X-succinimidyl ester labelled calmodulin and calcium as described in materials and methods. After that, we tested for the effects of various concentration ranges of unlabeled short cytoplasmic tail TM219 peptide on the dissociation of the labelled calmodulin from the TM219 nanodisc tetrapartate complex at room temperature for 30 min. As the concentration of the free peptide increased, the changes in FP signal decreased. The free peptide competed with the TM219 receptor binding to calmodulin with a calculated inhibition constant (Ki) ~23 nm. **B**- MCF7 cells treated with pHLIP-cy3 peptide at pH 7.4 or pH 5.5 for 5 minutes. Cells were fixed and examined with fluorescence microscopy. Staining of the cells with red dye indicates insertion of pHLIP-cy3 into the cell membrane of MCF7 cells. Nuclei were stained with Hoechst dye (blue). **C**-MCF7 cells pretreated with either SCTT-pHLIP or pHLIP peptide alone were treated with 1μM IGFBP3 for 1hour in DMEM serum free medium. Cell lysates were subjected to immunoblot using anti-human phosphor-beclin1 antibody. Treatment with SCTT-pHLIP reduced drastically phosphorylation of Beclin1 protein.

Studies were next undertaken to determine if a peptide with the sequence of the short cytoplasmic tail of TM219 (SCTT) could interfere with the binding of TM219 to calmodulin in the presence of calcium and IGFBP3. This was done with the tetrapartite complex of TM219 nanodisc, IGFBP3, calcium and BODIPY-TRM-X-succinimidyl ester labelled calmodulin. The components of this complex were mixed in the presence of the SCTT peptide as described in Materials and Methods, and binding was assessed. As the concentration of the free peptide increased, the changes in Fl. anisotropy signal decreased, indicating that the free peptide competed with the TM219 protein and sequestered the labelled calmodulin (Fig 6A). We used this inhibition plot in estimation of the inhibition constant (Ki) of the free peptide (~23 nM).

### Appropriately delivered SCTT peptide blocks TM219 mediated effector responses

In our early experiments, we pretreated Vero cells with different doses of the short cytoplasmic domain of TM219 peptide to study its effect on blocking autophagy mediated by IGFBP3-TM219 signaling module. We used Western blot hybridization to judge the difference in LC3 processing. There was no notable difference in processing of LC3 in IGFBP3 treated cells in presence or absence of the peptide (S5 Fig). We then used the biotinylated form of the SCTT peptide to trace its inside these cells. These studies demonstrated that the peptide accumulated in the endocytic pathway or other membranous compartment rather than the cytoplasm of the cells (S5 Fig). These negative data suggested that the failure of the SCTT peptide to inhibit autophagy might be because of its inability to access the cytoplasm. To address this possibility, we fused our peptide with an optimal cargo delivery system known as pH (low) insertion peptide (pHLIP) [61]. pHLIP is a soluble peptide derived from the bacteriorhodopsin transmembrane region. Under acidic condition, pHLIP peptide get inserted into the plasma membrane and delivers a C-terminal conjugated cargo into the cytoplasm of the recipient cells [61–64]. To establish this system, we first labeled the N-terminal of pHLIP peptide with Cy3 dye to assess its insertion into the plasma membrane under acidic condition. We used in this experiment MCF7 cells and highlighted the insertion of the peptide into the membrane when the pH of the culture medium was dropped (Fig 6B). We then used pHLIP-SCTT peptide to test for its inhibition effects on the signaling of IGFBP3 mediated by TM219 in MCF7 cells. As a readout assay, we used phosphorylation of Beclin1 protein. Cells treated with IGFBP3 and pHLIP-SCTT peptide demonstrated drastic reduction of Beclin1 phosphorylation compared to the control (Fig 6C). These studies demonstrated that SCTT inhibits TM219-IGFBP3 signaling when appropriately delivered into the cytoplasm.

### SCTT peptide inhibits the growth of highly metastatic breast cancer cells in 3D culture

**Fig 7:**
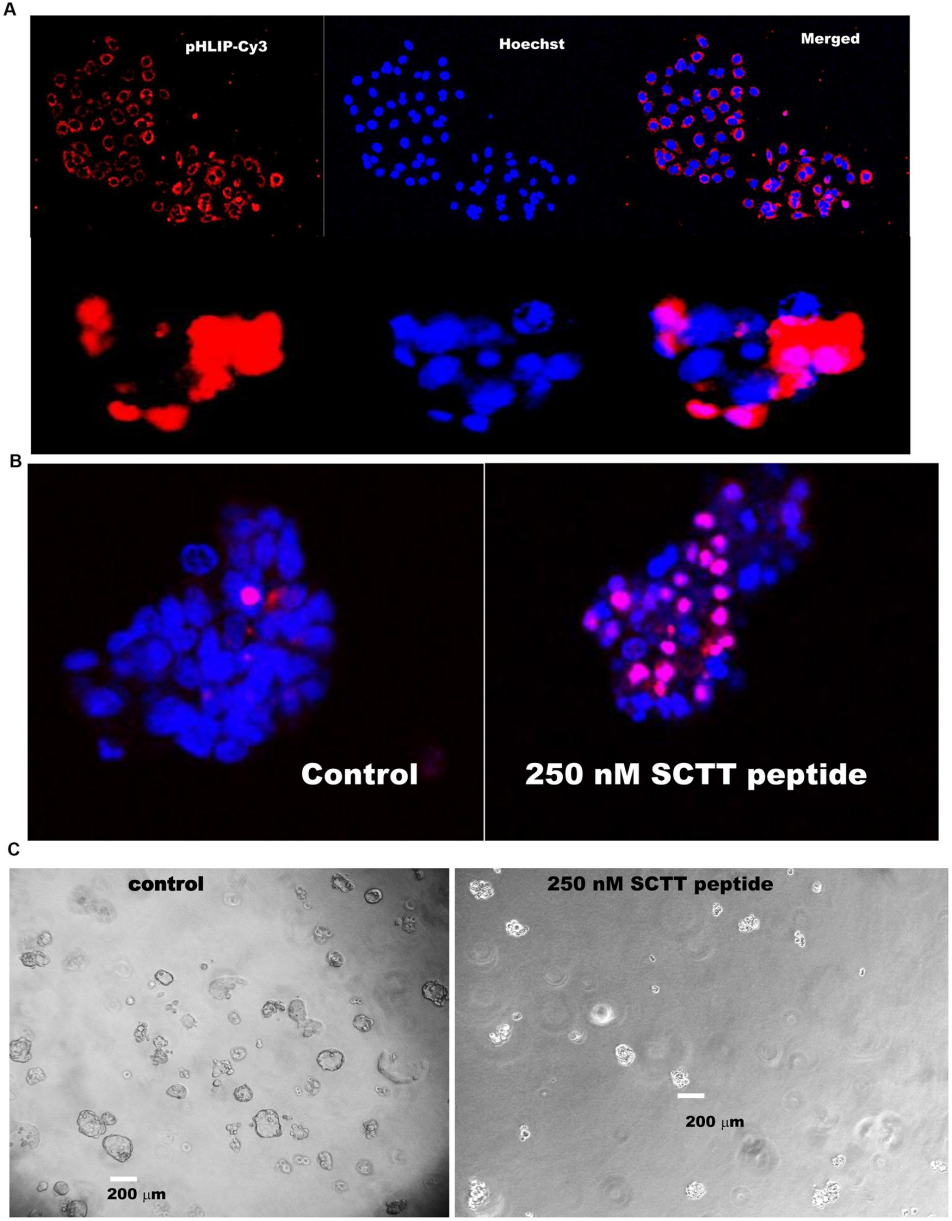
SCTT peptide inhibits the growth of highly metastatic breast cancer cells in 3D culture. **A**- MDA-MB231 cells were grown in a complete DMEM medium for 3 days or in 0.5% low melting agarose in complete medium for 5 days were treated with phLIP-Cy3 peptide in DMEM buffered with PBS for 6 hour at 37°C. Growth media were replaced with completed DMEM medium buffered with carbonate. Cells were imaged for insertion of the peptide. Red color indicates the insertion of pHLIP-cy3 peptide into the plasma membrane of MAD-MB231 in both two dimension and three dimension cell cultures. Nuclei of the cells were stained with Hoechst stain (blue) **B**-Cells pretreated with pHLIP-TM219 peptide or pHLIP peptide alone for 5 days in 0.5% low melting agarose in DMEM complete media supplemented with 1 μM IGFBP3. The peptides were mixed with DMEM buffered with PBS and added to the spheroids grown in low melting agarose. After 6 hours, the media were replaced with completed DMEM media buffered with carbonate pH 7.2. Cells were incubated for additional 9 days at 37°C in a CO2 incubator. Cells pretreated with SCTT peptide demonstrated significant reductions in their size and higher number of dead cells as judged by inclusion of propodeum Iodide (PI, Red) relative to cells pretreated with pHLIP peptide alone. Living cells were assessed by Hoechst dye stain (Blue). **C**-MDA-MB231 spheroids were grown for 14 days of growth in 0.5% low melting agarose in presence or absence of SCTT peptide. Spheroids treated with the SCTT peptide demonstrated a drastic reduction of their sizes compared to MDA-MB231 control spheroids treated with pHLIP peptide alone.

MDA-MB231 are triple negative highly metastatic human breast cancer cells commonly used to study breast cancer metastasis and the therapy outcome [1]. To assess the efficacy of the SCTT-pHLIP system and the role of TM219 in the phenotype of these cells, we employed 3D culture of MDA-MB231 [49]. We first tested for the delivery of pHLIP-cy3 peptide to MDA-MB213 in two dimensional and three dimensional cell cultures (Fig 7A, upper and lower panels respectively). We then used this system to study the effects of pHLIP-SCTT peptide on the cell growth and survival after treatment with IGFBP3. MDA-MB231 spheroids pretreated with IGFBP3 followed by treatment with pHLIP-SCTT peptide significantly increased the number of dead cells and reduced the size of the spheroids relative to the control (Fig 7B and 7C, Table 2).

**Table 2:** Some changes in MDA-MB231 spheroids after treatment with SCTT peptide.

	Radius(μm)	Av. Vol.(μm3)	Ave. no. of cells/spheroids	Viability%
MDA-MB231	93+/-12	3369282	315+/-30	94
MDA-MB231+ SCTT	48+/-5	463247	127+/-13	33

These data indicate that the synthetic short cytoplasmic tail peptide of TM219 is an effective tool to interrupt TM219-IGFBP3 signaling in 3D cell culture and can be used as an effective tool to restore normal cell death in therapy resistant cells.

## Discussion

TM219 receptor has been linked to different human diseases such as asthma and cancer [32], [37]. However, the mechanism of how this protein functions has been poorly addressed. The presence of the di-arginine motif (RR, RTR) in the cytoplasmic domain of TM219 suggested that this protein is expressed and retained in the ER compartment. The mechanism of di-arginine motif retention and how membrane proteins with such motif exit the ER compartment has been studied intensively in similar membrane proteins [50–51], [65–68]. Consistent with our observation that TM219 is expressed and retained in ER compartment; TM219-eGFP expression and immunostaining data of TM219 indicated that it is mainly localized in the perinuclear region. Moreover, our biochemical approaches to define proteins that interact with TM219 short cytoplasmic tail identified different proteins that are involved in ER retention and exist of similar membrane proteins.

TM219-IGFBP3 signaling module was proposed to activate processing of procaspase-8 [32], [37]. However, TM219 protein has a truncated cytoplasmic domain (17 amino acids) that cannot justify for formation of any known cell death activation domains such as caspase activating domains (CARD), death domains or death like domains. Previous studies also did not explain how TM219 protein functions as a signaling receptor with 17 amino acids cytoplasmic tail [32], [47], [37]. We used biochemical approaches to identify calmodulin and calmodulin dependent kinase II (CaMKII). CaMKII is a multifunctional serine/threonine specific kinase that is activated via autophosphorylation upon binding to Ca^++^/calmodulin [69]. Blocking caMKII activity in human breast cancer MCF7 cells has been reported to augment their cell death [70]. Moreover, activated caMKII phosphorylates Beclin1 at serine residue 90 (Ser90) and induces autophagy [56]. Calmodulin is known to bind with high affinity to a relatively small alpha-helical region of many proteins in either calcium dependent or independent manner [71]. We confirmed the binding of calmodulin to TM219 *in vivo* and *in vitro*. This binding required the presence of IGFBP3 protein and calcium *in vitro*. Intracellular calcium elevation after IGFBP3 treatment has been reported previously in MCF7 [52], our data indicated that this effect is independent of TM219 receptor. These findings suggested that IGFBP3 activates caMKII protein via two different receptors; unknown receptor that triggers an increase in intracellular calcium and TM219 receptor.

Another interesting protein that we found in our pull-down experiment using TM219 short cytoplasmic tail is the ER molecular chaperon GRP78, also known as Bip. ER molecular chaperons such as GRP78 and calcium binding protein calreticulin activate autophagy during ER stress in response to unfold protein accumulation [72–73]. They recognize the hydrophobic batch of the nascent polypeptide in ER lumen. Failure of folding the newly translated polypeptide results in activation of autophagy and degradation of the ER unfolded proteins. In IGFBP3-TM219 signaling module mediated autophagy, IGFBP3 ligand, not the ER unfolded polypeptide, binds directly to TM219 and activates autophagy. The role of GRP78 in TM219 signaling pathways and whether it is interacting directly with TM219 protein or associated with other proteins interacting with TM219 will be the focus of another study.

Finally, we used a rational design approach to interrupt TM219 downstream signaling by testing the hypothesis that a free peptide based on the sequence of the cytoplasmic tail of TM219 protein (SCTT) will compete with TM219 receptor binding to calmodulin or other downstream effectors and therefore will block the downstream signaling of this pathway. We first calculated the inhibition constant of the peptide (Ki ~23 nM) using a competition binding assay of TM219 nanodisc and fluorescence anisotropy assay. Then, we fused SCTT peptide with pHLIP peptide to ensure its delivery to the cytoplasm of the recipient cells. In addition to known advantage pHLIP peptide in targeting the acidic micro-environment of cancer cells, no toxicity or membrane leakage effects of this peptide at neutral or low pH has been reported [61], [74–75]. Targeting SCTT peptide to the cytoplasm of breast cancer cells results in drastic reduction of phosphorylation of Beclin1 and reducing the size of spheroids.

In summary, we used *in vivo* and *in vitro* approaches to study and block the signaling of TM219 receptor. Our data identified a novel function of TM219 protein mediated by IGFBP3 as an activator of autophagy. They also identified calcium, calmodulin, and caMKII as additional components of the IGFBP3-TM219 signaling complex. This signaling complex can be inhibited by a synthetic peptide derived from the short cytoplasmic tail peptide of TM219 when appropriately delivered to the cytoplasm of the recipient cells.

### Limitation of the study

The mechanism of how TM219 protein assembles and exits ER to the cell surface is out of the scope of this study and requires further studies. Also, a further study is required to evaluate the effects of SCTT peptide in a human breast cancer animal model.

## Supporting information

S1 Fig TM219 protein is co-localized with the perinuclear compartment protein makers. Vero cells were co-transfected with TM219-GFP fusion and the lysosomal protein Lamp1-monomeric red fluorescence (Lamp1-mRFP) (upper panel). Cells were fixed and examined with fluorescence microscopy. Partial overlapping of TM219-GFP fusion was detected with Lamp1 protein. We also overexpressed TM219-GFP fusion and feed the grown cells with the intact mitochondrial dye TMRM. Cells were fixed and examined with fluorescence microscopy. Stained mitochondria overlapped with TM219-GFP expression.
(TIF)

S2 Fig IGFBP3-TM219 signaling module induces autophagy in Vero cells. **A**- Vero cells transfected for 48 hours with eGFP-TM219 manifest clearly perceptible large internal vacuoles relative to cells transfected with eGFP alone. Cells also were healthy with intact nuclei. Hoechst dye was used to stain the nuclei (blue). B-Vero cells transfected for 48 hours with eGFP-TM219 activates higher level of processing of LC3 protein compared to cells transfected with eGFP alone. The relative quantification ratio between LC3-II and LC3-I was measured using ImageJ software as described in materials and methods. Probing the lysate with anti- procaspase-3 antibody did not indicate activation of programmed cell death pathways.
(TIF)

S3 Fig Cloning, expression and purificaiton of human calmodulin. **A**- Human calmodulin was amplified and cloned from total RNA isolated from Thp1 cells. The protein was expressed in Rosetta strain of *E*.*coli* and purified first based on its hydrophobicity using phenyl sepharose column as described in materials and methods. Different fractions were eluted with 1 mM EGTA, resolved on 4–20% dPAGE and stained with Coomassie dye. **B**-Combined fractions eluted from phenyl sepharose column were subjected to monoQ column purification. Protein was eluted with a gradient concentration of 0–100% Nacl in 10 mM Tris pH 7.4, resolved on 4–20% dPAGE and stained with Coomassie dye. **C**-After monoQ column, protein was subjected to size exclusion chromatography using S200 column. Since the amino acid sequence of human calmodulin does not contain tryptophan, we used dPAGE and Coomassie dye to monitor the eluted protein. Positive factions were concentrated using 10KD ultrafiltration tube, resolved on 4–20% SDS dPAGE and stained with Coomassie dye.
(TIF)

S4 Fig Calmodulin and IGFBP3 bind to TM219 nanodisc specifically. **A**-The empty nanodisc (0–2000 nM) was used to test for its binding to labelled IGFBP3. No specific binding was detected. **B**-TM219 nanodisc (0–100 μM) was used to test for its binding to the labelled calmodulin in presence of 1μM IGFBP3. No specific binding was detected. **C**-TM219 nanodisc (0–100 μM) was used to test for its binding to the labelled calmodulin in presence of calcium and in absence of IGFBP3. No specific binding was observed. **D**-Empty nanodisc (0–100 μM) was used to test for its binding to calmodulin in presence of 1mM calcium chloride and 1 μM IGFBP3. No specific binding was observed.
(TIF)

S5 Fig Treatment with the short cytoplasmic tail of TM219 does not block autophagy. **A**-Different doses (0, 25, 250 nM) of the short cytoplasmic tail of TM219 peptide was used to treat Vero cells in DMEM serum free medium for 1 hour in presence of 1 μM of IGFBP3 protein. Lysates were immunoprobed with anti-LC3 and anti-β-actin. The relative quantification ratio between LC3-II and LC3-I was measured using ImageJ software as described in materials and methods. **B**-Vero cells were treated with the biotinylated TM219 peptide for 1 hour in presence of IGFBP3 and examined with the fluorescence microscopy as described in materials and methods. Cells treated with the biotinylated form of the TM219 short cytoplasmic tail peptide showed a clear red signal (streptavidin labelled Alex5559) accumulated in an intracellular membranous compartment. Hoechst dye was used to stain the nuclei (blue).
(TIF)
