# Supplementary figures and images for: Blocking transmembrane219 protein signaling inhibits autophagy and restores normal cell death
Source: PLoS One. 2019 Jun 20;14(6):e0218091. doi: 10.1371/journal.pone.0218091 (PMC6586287; doi:10.1371/journal.pone.0218091)

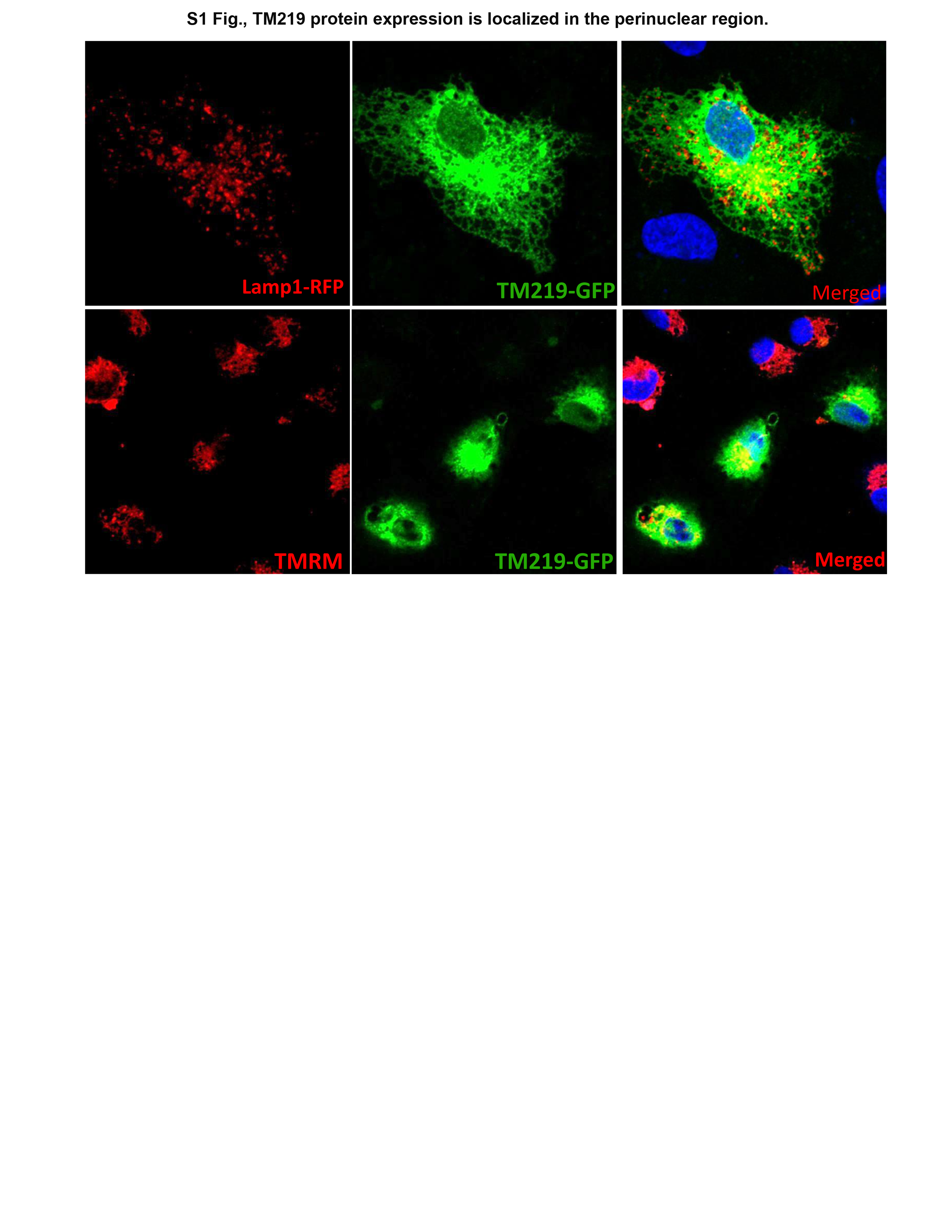

Supplement: S1 Fig — Vero cells were co-transfected with TM219-GFP fusion and the lysosomal protein Lamp1-monomeric red fluorescence (Lamp1-mRFP) (upper panel). Cells were fixed and examined with fluorescence microscopy. Partial overlapping of TM219-GFP fusion was detected with Lamp1 protein. We also overexpressed TM219-GFP fusion and feed the grown cells with the intact mitochondrial dye TMRM. Cells were fixed and examined with fluorescence microscopy. Stained mitochondria overlapped with TM219-GFP expression. (TIF) [file pone.0218091.s001.tif]

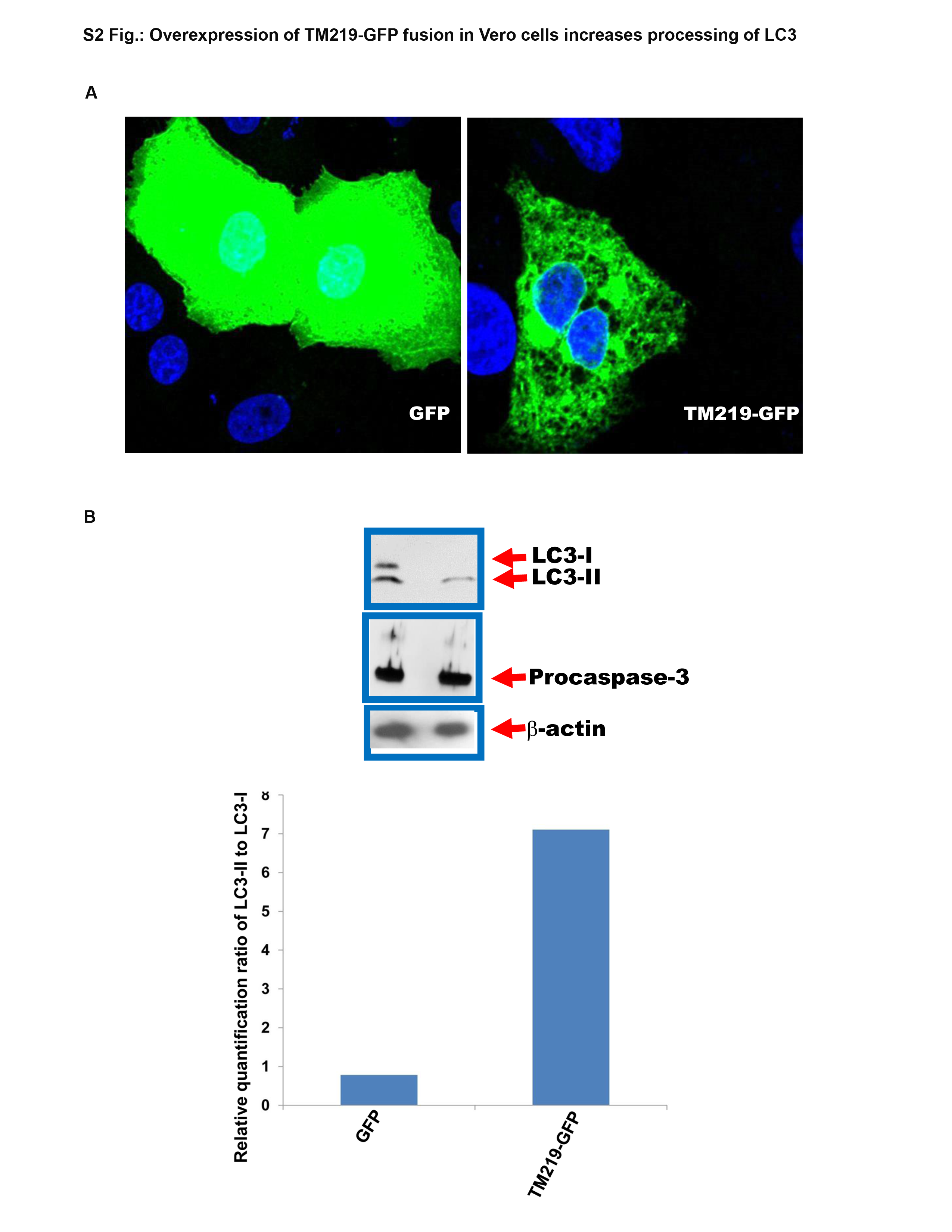

Supplement: S2 Fig — A- Vero cells transfected for 48 hours with eGFP-TM219 manifest clearly perceptible large internal vacuoles relative to cells transfected with eGFP alone. Cells also were healthy with intact nuclei. Hoechst dye was used to stain the nuclei (blue). B-Vero cells transfected for 48 hours with eGFP-TM219 activates higher level of processing of LC3 protein compared to cells transfected with eGFP alone. The relative quantification ratio between LC3-II and LC3-I was measured using ImageJ software as described in materials and methods. Probing the lysate with anti- procaspase-3 antibody did not indicate activation of programmed cell death pathways. (TIF) [file pone.0218091.s002.tif]

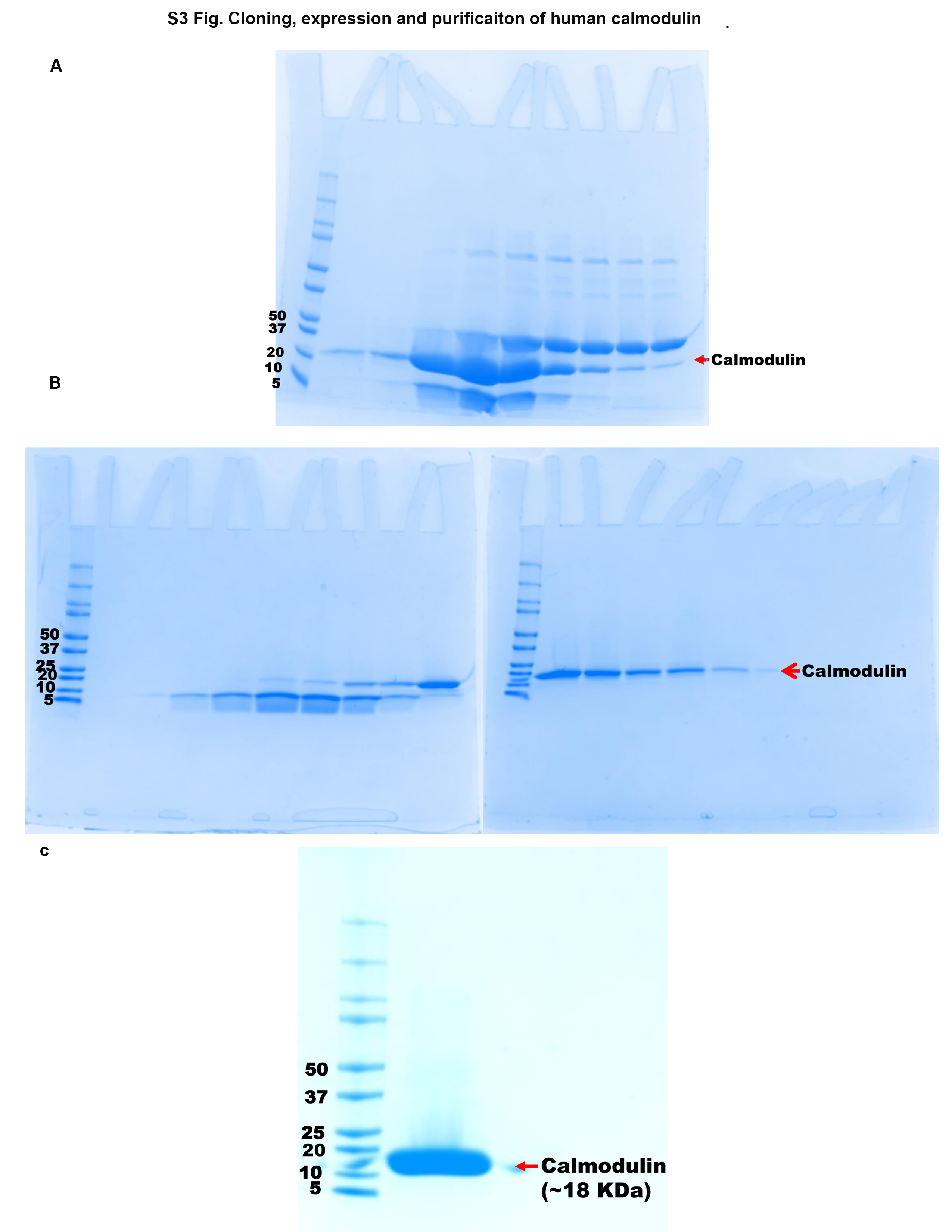

Supplement: S3 Fig — A- Human calmodulin was amplified and cloned from total RNA isolated from Thp1 cells. The protein was expressed in Rosetta strain of E.coli and purified first based on its hydrophobicity using phenyl sepharose column as described in materials and methods. Different fractions were eluted with 1 mM EGTA, resolved on 4–20% dPAGE and stained with Coomassie dye. B-Combined fractions eluted from phenyl sepharose column were subjected to monoQ column purification. Protein was eluted with a gradient concentration of 0–100% Nacl in 10 mM Tris pH 7.4, resolved on 4–20% dPAGE and stained with Coomassie dye. C-After monoQ column, protein was subjected to size exclusion chromatography using S200 column. Since the amino acid sequence of human calmodulin does not contain tryptophan, we used dPAGE and Coomassie dye to monitor the eluted protein. Positive factions were concentrated using 10KD ultrafiltration tube, resolved on 4–20% SDS dPAGE and stained with Coomassie dye. (TIF) [file pone.0218091.s003.tif]

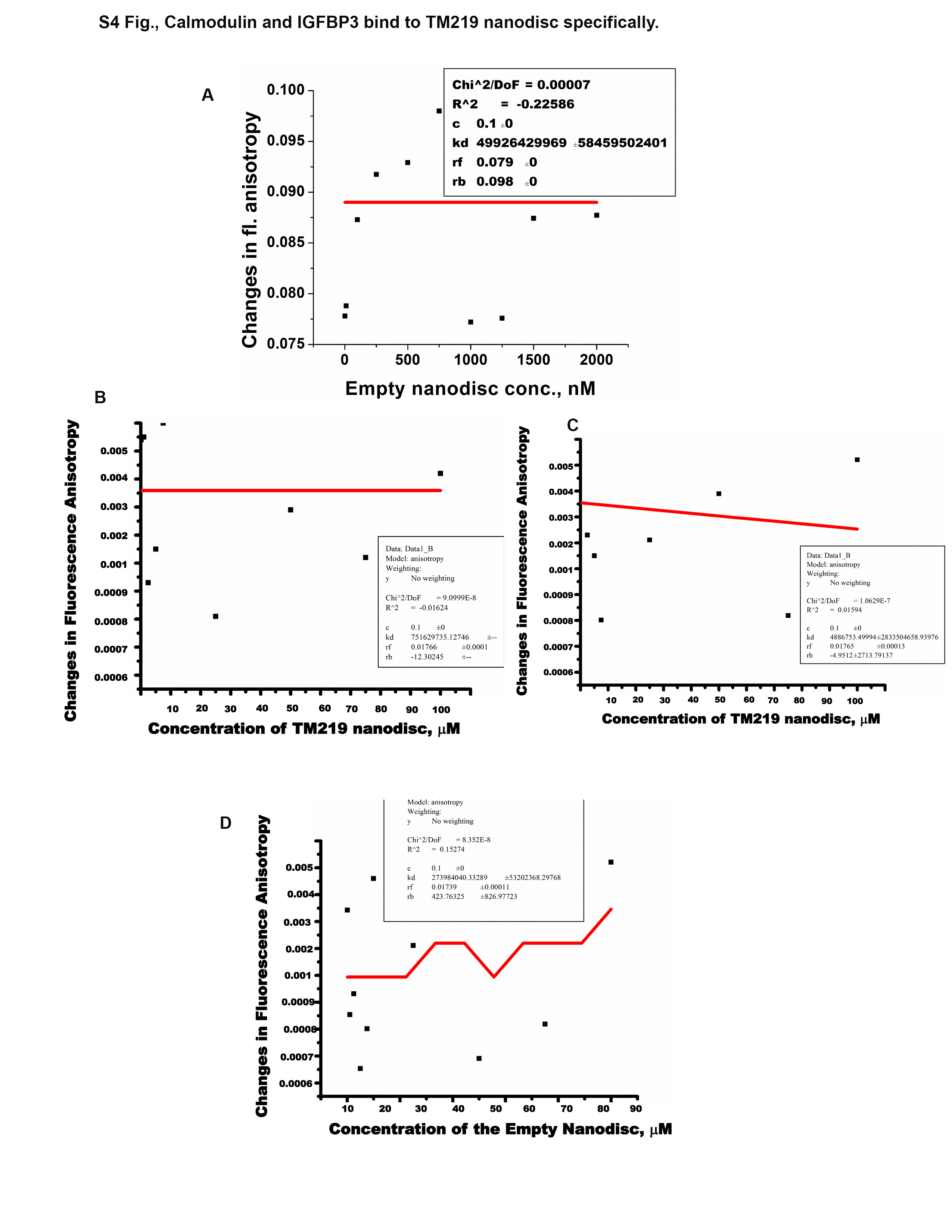

Supplement: S4 Fig — A-The empty nanodisc (0–2000 nM) was used to test for its binding to labelled IGFBP3. No specific binding was detected. B-TM219 nanodisc (0–100 μM) was used to test for its binding to the labelled calmodulin in presence of 1μM IGFBP3. No specific binding was detected. C-TM219 nanodisc (0–100 μM) was used to test for its binding to the labelled calmodulin in presence of calcium and in absence of IGFBP3. No specific binding was observed. D-Empty nanodisc (0–100 μM) was used to test for its binding to calmodulin in presence of 1mM calcium chloride and 1 μM IGFBP3. No specific binding was observed. (TIF) [file pone.0218091.s004.tif]

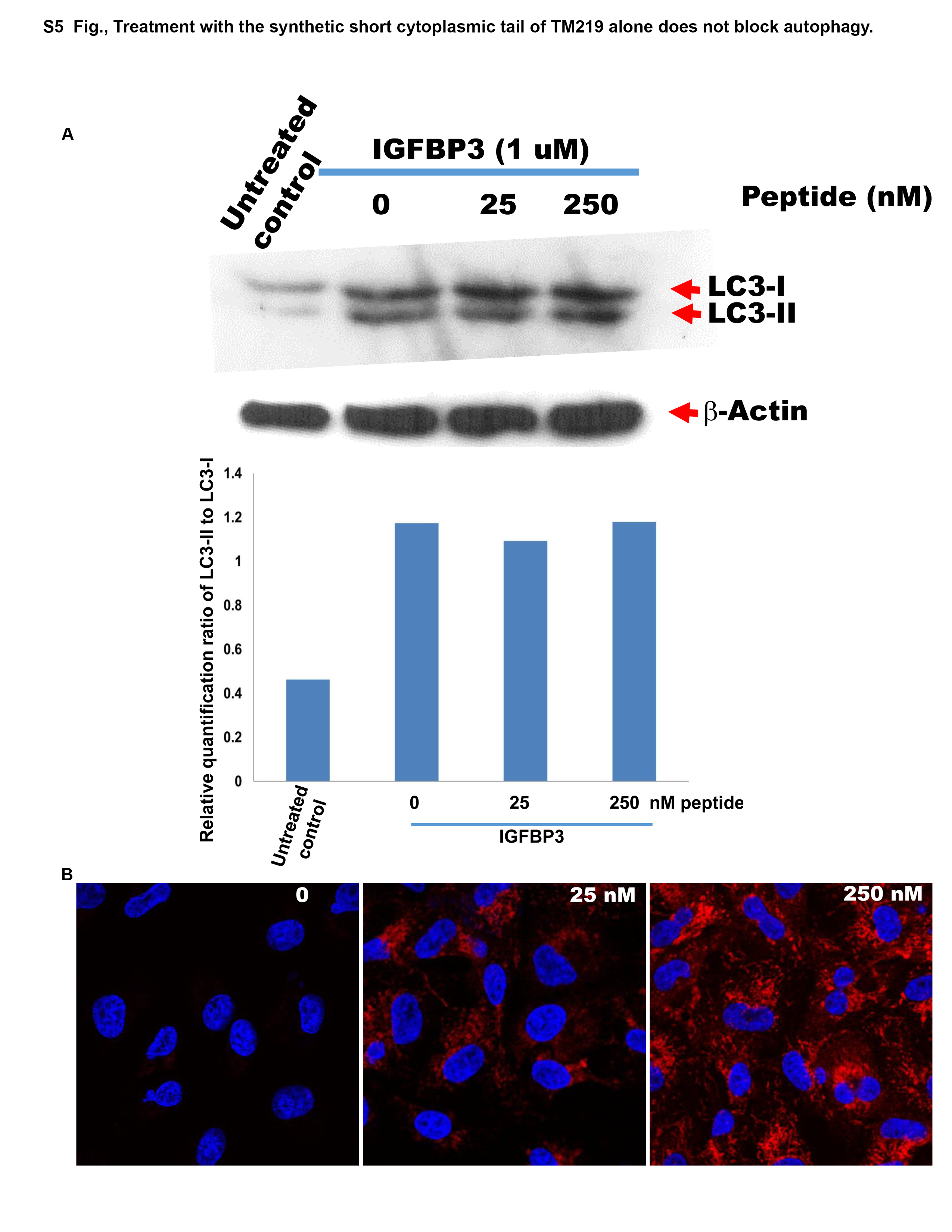

Supplement: S5 Fig — A-Different doses (0, 25, 250 nM) of the short cytoplasmic tail of TM219 peptide was used to treat Vero cells in DMEM serum free medium for 1 hour in presence of 1 μM of IGFBP3 protein. Lysates were immunoprobed with anti-LC3 and anti-β-actin. The relative quantification ratio between LC3-II and LC3-I was measured using ImageJ software as described in materials and methods. B-Vero cells were treated with the biotinylated TM219 peptide for 1 hour in presence of IGFBP3 and examined with the fluorescence microscopy as described in materials and methods. Cells treated with the biotinylated form of the TM219 short cytoplasmic tail peptide showed a clear red signal (streptavidin labelled Alex5559) accumulated in an intracellular membranous compartment. Hoechst dye was used to stain the nuclei (blue). (TIF) [file pone.0218091.s005.tif]
